# Efficacy of Credelio Quattro™ (lotilaner, moxidectin, praziquantel, and pyrantel chewable tablets) and Credelio™ (lotilaner) chewable tablets in the prevention of *Borrelia burgdorferi* transmission from infected *Ixodes scapularis* in dogs

**DOI:** 10.1186/s13071-025-07136-9

**Published:** 2025-12-06

**Authors:** William Anderson, Molly D. Savadelis, Scott Wiseman, Abdelmoneim Mansour, Riaan Maree, Lisa Young

**Affiliations:** 1 Elanco Animal Health, Innovation Way, Indianapolis, IN 46221 USA; 2 Elanco Animal Health, Bartley Way, Hook, RG27 9XA UK; 3 TRS Labs Inc, Athens, GA 30607 USA; 4 Clinvet US, Waverly, NY 14892 USA

**Keywords:** Credelio Quattro, Lotilaner, Credelio, *Borrelia burgdorferi*, Vector-borne diseases

## Abstract

**Background:**

*Borrelia burgdorferi*, the causative agent of Lyme disease, is a zoonotic vector-borne pathogen transmitted by various *Ixodes* tick species. Lyme disease, while commonly asymptomatic, can induce fever and intermittent lameness in dogs. Highly effective acaricidal products with a rapid onset of action along with prompt removal of attached ticks are important aspects of successful Lyme disease prevention strategies.

**Methods:**

Two studies were conducted with a total of 30 dogs each. Dogs were randomized to receive a control sham dose, Credelio Quattro, or Credelio. Treatment was administered on Day 0 in a fed state. On Day 28, all dogs were experimentally infested with wild-caught adult *Ixodes scapularis*. Blood samples for *B. burgdorferi* antibody analysis utilizing the SNAP 4Dx Plus and Lyme Quant C_6_ tests were collected on Days 27, 49, 63, 77, 91, and 105. Skin biopsies were collected from four different areas of heavy tick attachment from each dog for polymerase chain reaction (PCR) detection of *B. burgdorferi* on Day 104 or 105.

**Results:**

All control dogs demonstrated adequate *I. scapularis* infestation rates on Day 33 in both studies. In Study 2, on Day 27, one control dog tested positive for *B. burgdorferi* on the Lyme Quant C_6_ test, prior to experimental tick infestation, and therefore was excluded from analysis. A total of eight out of 10 (Study 1) and nine out of nine (Study 2) control dogs tested positive for *B. burgdorferi* on at least one test after Day 27. One dog in the Credelio Quattro-treated group tested positive for *B. burgdorferi* on SNAP 4Dx Plus on Day 105 in Study 1 but was negative on all other tests and study days. None of the dogs treated with Credelio tested positive for *B. burgdorferi* at any point during either study.

**Conclusions:**

The laboratory studies described herein confirm that a single dose of lotilaner, at the minimum effective dosage of 20 mg/kg, administered as Credelio Quattro, in combination with moxidectin, praziquantel, and pyrantel or Credelio, is effective for the prevention of transmission of *B. burgdorferi* from infected *I. scapularis* for a full month in dogs.

**Graphical Abstract:**

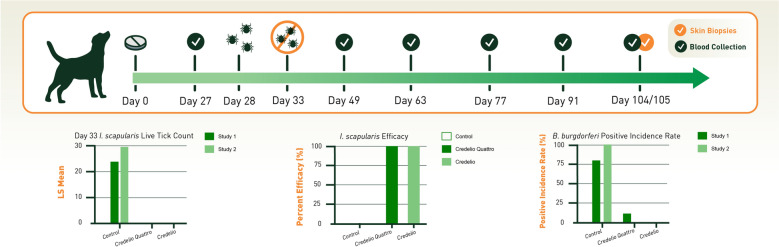

## Background

The causative agent of Lyme disease, *Borrelia burgdorferi*, is a bacterial vector-borne pathogen that can affect both humans and animals. Transmission occurs through various *Ixodes* tick species (e.g., *Ixodes scapularis*, *I. ricinus*, *I. hexagonus*, *I. pacificus*, and *I. persulcatus*) across North America, Europe, and parts of Asia [1]. Lyme disease incidence continues to rise globally, driven by factors such as expanding tick habitats, increased overlap between human, animal, and tick environments, and climate change [2–5]. In the United States, Lyme disease incidence in humans is highest in the New England and Mid-Atlantic regions, followed by the South Atlantic and North Central regions [3, 6]. In Europe, incidence is widespread throughout due to the high distribution of *I. ricinus* [3]. Despite regional differences in incidence, all dogs are potentially at risk of tick infestations and *B. burgdorferi* transmission due to increasing owner and pet travel and movement of rescue dogs nationally.

Transmission of *B. burgdorferi* to *Ixodes* spp. occurs when tick larvae feed on infected rodents such as the white-footed mouse, *Peromyscus leucopus*, ingesting spirochetes attracted to the feeding site by chemotactic signals [7, 8]. Throughout the tick molting process from larvae to nymph post-feeding, spirochetes remain in the lumen of the tick gut, migrating to the salivary glands of nymphal stages during blood-feeding thereafter [9]. While both nymphal and adult stages of *Ixodes* are capable of transmitting *B. burgdorferi*, the highest risk of transmission occurs with nymphs due to their high population numbers and small size as compared with adult stages [10]. Nymphs are typically most actively questing in early spring through summer, feeding on mammals including dogs, horses, and humans [11].

Common clinical signs of Lyme disease in dogs include fever, anorexia, lethargy, swollen lymph nodes, intermittent shifting lameness, and potentially Lyme glomerulonephritis, which can be fatal [12, 13]. While many dogs infected with *B. burgdorferi* are asymptomatic, humans are more likely to present with clinical symptoms [12, 14]. Within a few days of the tick bite, many people develop a bull’s eye rash, erythema migrans, at the bite site along with non-specific flu-like symptoms [15]. In the early disseminated phase, within weeks of the infected tick bite, symptoms can include facial palsy, meningitis, joint inflammation, or cardiac irregularities [16]. Months after the infected tick bite, in the late disseminated phase, symptoms such as intermittent arthritis, severe joint swelling, and neurological dysfunctions have been reported [15, 17].

Treatment for Lyme disease in both dogs and humans includes antibiotics such as doxycycline to eliminate *B. burgdorferi* spirochetes and clinical management as needed [14, 18]. Antibiotic treatment can be extended longer than the recommended 4-week course or repeated as needed to achieve clinical resolution [18]. Despite treatment, some people continue to experience clinical symptoms of lethargy, joint pain, or neurological dysfunctions, termed post-treatment Lyme disease syndrome, highlighting the importance of reducing tick exposure and risk of disease transmission [19].

Reducing the risk of disease transmission for *B. burgdorferi* centers around decreasing tick exposure and bites. Effective preventive strategies for decreasing tick exposure include avoiding tick habitats such as wooded areas and leaf litter, tick checks after potential exposure, prompt removal of ticks as soon as observed, and appropriate use of repellants and/or acaricidal products [20]. Transmission of *B. burgdorferi* has been documented to typically occur between 48 and 72 h after tick attachment [21] but has been demonstrated as early as 6 h in an in vitro study [22] and within 12–24 h in mice infested with *Borrelia*-infected nymphs or adult ticks, respectively [23]. It is therefore imperative to perform daily tick checks with immediate removal, along with the use of acaricidal products providing a rapid onset of action to significantly reduce transmission of vector-borne pathogens when tick exposure occurs [24–26].

Isoxazoline ectoparasiticides have demonstrated robust efficacy against flea and tick infestations on dogs. Most recently, the potential of isoxazolines in the prevention of tick-borne disease has been exhibited in dogs by killing ticks and preventing the transmission of vector-borne pathogens [27–30]. Lotilaner, an isoxazoline parasiticide, is administered orally as a chewable tablet and works systemically. Lotilaner (in Credelio, Credelio™ PLUS, Credelio Quattro, Elanco Animal Health, Indianapolis, IN, USA) is approved for use against ectoparasites in dogs. The objective of the studies described below was to evaluate the efficacy of lotilaner in the prevention of *B. burgdorferi* transmission from infected *I. scapularis* to dogs by killing the ticks before transmission could occur. This evaluation included lotilaner as a component of the novel chewable tablet Credelio Quattro (in combination with moxidectin, praziquantel, and pyrantel), and as the only active ingredient in Credelio.

## Methods

Two studies were conducted to evaluate the efficacy of Credelio Quattro and Credelio for the prevention of *B. burgdorferi* transmission from infected *I. scapularis* wild-caught adult ticks. All studies were conducted according to VICH [International Cooperation on Harmonization of Technical Requirements for Registration of Veterinary Medicinal Products] Guideline 9 (GL9) Good Clinical Practice, World Association for the Advancement of Veterinary Parasitology (WAAVP) guidelines for evaluating the efficacy of parasiticides for the treatment, prevention, and control of flea and tick infestations on dogs and cats, and WAAVP guidelines for studies evaluating the efficacy of parasiticides in reducing the risk of vector-borne pathogen transmission in dogs and cats [25, 26]. The protocols were reviewed and approved by the study site’s Animal Care and Use Committee prior to initiation.

### Animals

A total of 30 purpose-bred Beagles, 10 dogs per treatment group, ranging from 11 to 13 months of age and weighing between 5.3 and 11.5 kg, were enrolled in each study. To be eligible for inclusion, dogs were required to be healthy, not pregnant, lactating, or intended for breeding during the course of the study, and to test negative for *B. burgdorferi* antibodies on SNAP^®^ 4Dx^®^ Plus (IDEXX Laboratories, Westbrook, ME, USA) during acclimation. Throughout the studies, age-appropriate, commercially available diets and access to potable water ad libitum were provided. Study dogs were individually housed and provided appropriate enrichment items. Housing was environmentally controlled, and a photoperiod of approximately 12 h light and 12 h darkness was maintained by overhead fluorescent lamps.

### Randomization and treatment

On Day −3, study dogs were randomized according to a completely randomized design to receive a control sham dose, Credelio Quattro, or Credelio. Treatments were administered orally on Day 0 to dogs in a fed state, utilizing body weights collected on Day −7 or Day −4. Dogs received tablets to achieve as close as possible to the minimum target dosages of 20 mg/kg lotilaner, 0.02 mg/kg moxidectin, 5 mg/kg praziquantel, and 5 mg/kg pyrantel (Credelio Quattro) or 20 mg/kg lotilaner alone (Credelio) without underdosing. Dogs in the control group were mock-dosed using either fingers or a pill administration device.

### Safety assessments

A physical examination was performed and general health assessments for all study animals were conducted at least once daily prior to dose administration. On the day of treatment, dogs were observed just prior to dose administration on Day 0 and at 1 , 2 , and 6 h post-dosing. General health assessments starting on Day 1 and continuing thereafter were performed at least twice daily. Any abnormal observation after dosing, whether or not considered to be product-related, was recorded as an adverse health event and followed until resolution. Concomitant therapies administered were recorded.

### Experimental infestation and tick counts

Wild-caught *I. scapularis* adults collected from Rhode Island (Study 1) and Connecticut (Study 2) were utilized for experimental infestations. Polymerase chain reaction (PCR) analysis was performed on a randomly sampled selection of ticks from both collected populations to confirm the presence of *B. burgdorferi*. Specifically, 20 ticks were analyzed from Study 1 and 96 ticks were analyzed from Study 2 using a published method [31]. On Day 28, all dogs were experimentally infested with ~50 unfed adult ticks (approximately equal distribution of males and females) according to study site standard laboratory procedures. On Day 33, all ticks were removed from each dog and categorized based on two criteria: attachment status (free or attached) and viability (live or dead).

### Serology

Blood samples were collected from all dogs prior to treatment administration, on Day 27 prior to experimental infestation, and on Days 49, 63, 77, 91, and 105. Detection of the presence of *B. burgdorferi* antibodies in the blood samples collected was evaluated qualitatively using the SNAP 4Dx plus test and quantitatively using the Lyme Quant C_6_^®^ test (IDEXX Laboratories, Inc., Westbrook, ME, USA).

### Skin biopsies

After tick removal on Day 33, hair from four separate areas with the highest number of attached ticks or with the most notable inflammation from attached ticks was shaved to identify sites for skin biopsies. Tick attachment sites, primarily the dorsal head, neck, and back, were shaved periodically throughout the remainder of the study to maintain the site location. For Study 1, skin biopsies were performed on all dogs on Day 105; for Study 2, half the dogs had skin biopsies performed on Day 104, and the remaining half were performed on Day 105. Two 4.0-mm skin biopsies from each of the four areas previously selected for each dog were collected according to study site standard laboratory procedures into a media-free container. For each dog, one biopsy from each of the four sites was analyzed for *B. burgdorferi* DNA using the PCR methodology described by Pahl et al. [32]. The second biopsy from each site was retained as a backup.

### Statistical analysis

All statistical analyses were performed using SAS version 9.4 (SAS Institute, Cary, NC, USA), with the individual dog being the experimental unit. Hypotheses were tested at a two-sided 0.05 level of significance.

Efficacy for Credelio Quattro and Credelio against *I. scapularis* ticks removed on Day 33 was determined using least squares means (LSM) calculated from a linear model (Study 1) or a linear mixed model (Study 2) utilizing live (free and attached) tick counts as the response and treatment as a fixed effect, comparing each treated group versus the control group. Dogs in Study 2 were housed in two rooms (dogs in Study 1 were housed in one room); the linear mixed model for Study 2 also included room as a random effect to reflect the experimental design. Percent efficacy for each treated group was then calculated as 100 × (LSM_control _− LSM_treated_)/(LSM_control_). Statistically significant differences between a treated group and the control group were determined from the treatment fixed effect in the model. Adequacy of infestation was defined as ≥ 25% retention of live ticks in at least six control dogs on Day 33. The mean infestation rate for *I. scapularis* in the control group was calculated as 100 × (total number of live ticks recovered/ total number infested).

The ability of Credelio Quattro and Credelio to prevent or block the transmission of *B. burgdorferi* through the rapid killing of ticks was assessed by comparing the proportion of dogs infected with *B. burgdorferi* at the end of the study in each treated group to that in the control group using Fisher’s exact test. Exact (Clopper–Pearson) 95% confidence intervals (CI) were calculated for the proportion of dogs infected with *B. burgdorferi* in each group at the end of the study. Dogs were considered infected with *B. burgdorferi* when a positive test result was obtained on any of the antibody or skin biopsy PCR tests after experimental infestation on Day 28. To be considered non-infected, dogs were required to test negative on all antibody and skin biopsy PCR tests at all time points evaluated after experimental infestation.

## Results

All dogs in the control group were infested with ≥ 25% live tick attachment rates, meeting adequacy of infestation guidelines as defined by WAAVP (Table 1).

**Table 1:** Day 33 adequacy of infestation in control treatment groups

Study no.	Treatment group	Number of dogs with adequate infestation (mean infestation rate_a_)
1	Control	10/10 (45.4%)
2	Control	10/10 (57.4%)

_a_ Mean infestation rate = (total number of ticks recovered/total number infested) × 100

No live attached ticks were recovered from any dog treated with either Credelio Quattro or Credelio on Day 33, demonstrating 100% efficacy against *I. scapularis* (Table 2). The difference in live tick counts was statistically significant (*p* < 0.0001, Table 2) when each of the Credelio Quattro- and Credelio-treated groups was compared separately to the control.

**Table 2:** Efficacy of Credelio Quattro and Credelio against *I. scapularis* based on Day 33 LS mean total live tick counts

Study no	Treatment group	LS mean live tick counts (% efficacy)	*P*-value (test statistic_a_)	Attached ticks on Day 33 (AM_b_, range)	
				Live	Dead
1	Control	22.7	–	22.7 (15–30)	0.0
	Credelio Quattro	0.0 (100%)	< 0.0001 (*t*_18_ = 15.38)	0.0	0.6 (0–3)
	Credelio	0.0 (100%)	< 0.0001 (*t*_18_ = 15.38)	0.0	0.5 (0–2)
2	Control	28.7	–	22.8 (21–24)	0.1 (0–1)
	Credelio Quattro	0.0 (100%)	< 0.0001 (*t*_17_ = 16.63)	0.0	0.6 (0–5)
	Credelio	0.0 (100%)	< 0.0001 (*t*_17_ = 16.63)	0.0	1.1 (0–4)

_a_ *t*-value and degrees of freedom, _b_ arithmetic mean

PCR analysis of wild-caught tick populations utilized for infestation confirmed *B. burgdorferi* infection rates of 70% and 38.5% for Studies 1 and 2, respectively. Interestingly, of the 98 ticks (46 males, 46 females) randomly selected for PCR analysis in Study 2, the percent infectivity of *B. burgdorferi* was slightly higher in male ticks (19 of 48 positive, 39.6%) than in female ticks (18 of 48 positive, 37.5%).

After dose administration, four dogs were reported to have diarrhea on Day 0—one control, two Credelio Quattro-treated, and one Credelio-treated dog—and all recovered on Day 1 without treatment. In Study 1, one dog in the Credelio Quattro-treated group was reported as slightly depressed with excessive drooling, dehydration, and vomiting 3 days post-treatment. This dog was treated with maropitant (Cerenia^®^, Zoetis, Girona, Spain) and subcutaneous fluids and recovered the following day. Limping or lameness was observed in one control dog (Study 2) 41 and 70 days after experimental infestation, one Credelio Quattro-treated dog (Study 1) 7 days after experimental infestation, and one Credelio-treated dog (Study 2) 2 days after experimental infestation. All dogs were treated with non-steroidal anti-inflammatory drugs (NSAIDs) and recovered.

A dog was considered infected with *B. burgdorferi* if any one of the diagnostic tests (SNAP 4Dx Plus, Lyme Quant C_6_ or skin biopsy PCR) was positive after Day 28. Combining diagnostic test results in each treatment group across all time points evaluated after experimental infestation, 70% and 100% *B. burgdorferi*-positive incidence rates were observed in control dogs on SNAP 4Dx Plus, and 80% and 100% positive incidence rates were observed on Lyme Quant C_6_ in the control groups of Studies 1 and 2, respectively (Table 3).

**Table 3:** Frequency summary of *Borrelia burgdorferi* antibody-positive test results over time

Study day	Study no.	Treatment group	Number of dogs per group	SNAP 4Dx Plus, *n* (%)	Lyme quant C_6_, *n* (%)
27	1	Control	10	0	0
		Credelio Quattro	10	0	0
		Credelio	10	0	0
	2	Control	10	0	1_a_ (10.0)
		Credelio Quattro	10	0	0
		Credelio	10	0	0
49	1	Control	10	0	1 (10.0)
		Credelio Quattro	10	0	0
		Credelio	10	0	0
	2	Control	9_a_	0	0
		Credelio Quattro	10	0	0
		Credelio	10	0	0
63	1	Control	10	3 (30.0)	3 (30.0)
		Credelio Quattro	10	0	0
		Credelio	10	0	0
	2	Control	9_a_	5 (55.6)	6 (66.7)
		Credelio Quattro	10	0	0
		Credelio	10	0	0
77	1	Control	10	1 (10.0)	6 (60.0)
		Credelio Quattro	10	0	0
		Credelio	10	0	0
	2	Control	9_a_	7 (77.8)	8 (88.9)
		Credelio Quattro	10	0	0
		Credelio	10	0	0
91	1	Control	10	5 (50.0)	7 (70.0)
		Credelio Quattro	10	0	0
		Credelio	10	0	0
	2	Control	9_a_	9 (100)	9 (100)
		Credelio Quattro	10	0	0
		Credelio	10	0	0
104/105	1	Control	10	7 (70.0)	8 (80.0)
		Credelio Quattro	10	1 (10.0)	0
		Credelio	10	0	0
	2	Control	9_a_	9 (100)	9 (100)
		Credelio Quattro	10	0	0
		Credelio	10	0	0
Overall	1	Control	10	7 (70.0)	8 (80.0)
		Credelio Quattro	10	1 (10.0)	0
		Credelio	10	0	0
	2	Control	9_a_	9 (100)	9 (100)
		Credelio Quattro	10	0	0
		Credelio	10	0	0

_a_Dog 20-DXV in the Study 2 control group tested positive on the Lyme Quant C_6_ test prior to wild-caught tick infestation on Day 28 and therefore was excluded from *B. burgdorferi* data analyses

Skin biopsy PCR results demonstrated 40% and 100% *B. burgdorferi* positive incidence rates in the control groups of Studies 1 and 2, respectively (Table 4).

**Table 4:** Day 104/105 skin biopsy PCR results for the detection of *B. burgdorferi*

Study	Study day	Dogs PCR positive for *B. burgdorferi*		
		Control	Credelio Quattro	Credelio
1	105	4 of 10	0 of 10	0 of 10
2	104/105	9 of 9_a_	0 of 10	0 of 10

_a_Dog 20-DXV in the Study 2 control group tested positive on Lyme Quant C_6_ test prior to wild-caught tick infestation on Day 28 and therefore was excluded from *B. burgdorferi* data analyses

One dog in the Credelio Quattro-treated group in Study 1 tested positive for *B. burgdorferi* on SNAP 4Dx Plus performed on Day 105, while all Lyme Quant C_6_ and skin biopsy PCR results for this dog tested negative on all time points. No dogs treated with Credelio tested positive for *B. burgdorferi* on any tests at any time points evaluated. Final infection rates in the control group demonstrated that eight of 10 dogs (80%) in Study 1 and nine of nine dogs (100%) in Study 2 tested positive for *B. burgdorferi* on any test at any time point (Table 5) post-infestation.

**Table 5:** Overall positive incidence rate for detection of *B. burgdorferi* for each study and treatment group

Study no.	Treatment group	Positive incidence *n*/*N* (%)	95% CI for positive incidence (%)_b_	*P*-value (test statistic_c_)
1	Control	8/10 (80)	(44.4, 97.5)	–
	Credelio Quattro	1/10 (10)	(0.3, 44.5)	0.0055 (*P* = 0.0027)
	Credelio	0/10 (0)	(0, 30.8)	0.0007 (*P* = 0.0004)
2	Control	9/9_a_ (100)	(66.4, 100)	–
	Credelio Quattro	0/10 (0)	(0, 30.8)	< 0.0001 (*P* < 0.0001)
	Credelio	0/10 (0)	(0, 30.8)	< 0.0001 (*P* < 0.0001)

_a_Dog 20-DXV in the Study 2 control group tested positive on Lyme Quant C_6_ test prior to wild-caught tick infestation on Day 28 and therefore was excluded from *B. burgdorferi* data analyses

_b_ 95% CIs are exact (Clopper–Pearson) 95% confidence intervals for proportions

_c_The test statistic *P* for Fisher’s exact test in parentheses is the “table probability” calculated using a hypergeometric distribution

For dogs treated with Credelio Quattro, the final infection rates were 1/10 dogs (10%) and 0/10 dogs (0%) in Studies 1 and 2, respectively, while no dogs treated with Credelio tested positive at any time point in either study. Differences in final positive infection rates between Credelio Quattro- and Credelio-treated groups versus control were statistically significant (*p* ≤ 0.0055).

## Discussion

While the precise timing of *B. burgdorferi* transmission after tick attachment in dogs is not fully understood, the recommendation to promptly remove ticks before they can transmit disease is crucial. A key strategy for reducing pathogen transmission from ticks is to use acaricidal products with a rapid speed of kill of < 24 h when tick exposure occurs. Lotilaner, from the isoxazoline class, is a potent antagonist of invertebrate γ-aminobutyric acid (GABA)-gated chloride channels [33]. The rapid speed-of-kill of lotilaner against *Amblyomma americanum* has been shown to be faster than other products containing members of the isoxazoline class [34]. Additionally, previous studies evaluating the speed-of-kill of 20 mg/kg lotilaner contained in Credelio and Credelio Quattro against *I. ricinus* demonstrated ≥ 84.6% efficacy 12 h post-infestation through 35 days after treatment administration [35]. This efficacy increased to ≥ 94.2% when live ticks removed 12 h post-infestation from lotilaner-treated dogs were evaluated after an additional 24-h incubation. Additionally, lotilaner was noted to begin killing *I. ricinus* as fast as 4 h after treatment administration, providing rapid onset of efficacy against pre-existing tick infestations [35]. The ability of lotilaner to provide rapid speed-of-kill against *Ixodes* spp. enabled the robust efficacy observed in these studies with Credelio Quattro and Credelio, preventing *B. burgdorferi* transmission from infected *I. scapularis* wild-caught ticks to dogs.

The wild-caught *I. scapularis* ticks, with a tick infectivity for *B. burgdorferi* of 70% in Study 1 and 38.5% in Study 2, demonstrated a robust challenge, as confirmed by the infections in the control group of each study. The infectivity rates shown in these studies are similar to what has been reported previously in these geographical areas for adult *I. scapularis* and are comparable to a recent publication using ticks from similar geographies [30].

Final infection rates in the control group confirmed a high infection rate in both studies, with 80% of dogs in Study 1 and 100% of dogs in Study 2 testing positive for *B. burgdorferi* on any test at any time point post-infestation. Despite this high diagnostic rate, only one dog in the control group was observed to have limping or lameness post-infestation, which was observed recurrently 41 and 70 days post-infestation. This observation is consistent with clinical signs of disease manifesting 2–5 months after exposure to a *B. burgdorferi*-infected tick [36]. One dog in each of the Credelio Quattro-treated group and the Credelio-treated group was observed to have limping or lameness 7 and 2 days post-infestation, respectively. Both dogs tested negative on all tests at all time points for *B. burgdorferi*, and therefore the observed clinical signs were likely not attributable to Lyme disease.

One dog in the Credelio Quattro-treated group tested positive for *B. burgdorferi* antibodies on SNAP 4Dx Plus on Day 105, most likely representing a false-positive result, as both Lyme Quant C_6_ and skin biopsy PCR results, also conducted on Day 105, tested negative. All other SNAP 4Dx Plus and Lyme Quant C_6_ evaluations for this dog tested negative on all other study days. The sensitivity and specificity of the SNAP 4Dx Plus test for the detection of *B. burgdorferi* antibodies were recently evaluated against North American field-sourced canine samples compared to a Western blot reference, demonstrating sensitivity and specificity of 80.7% (95% CI 70.6–88.6) and 92.8% (95% CI 89.1–95.5), respectively [37]. When analyzing SNAP 4Dx Plus *B. burgdorferi* results as compared with Western blot results, 21 samples were characterized as false-positive results, and 16 samples were false-negative results [29].

Preventing *B. burgdorferi* transmission is crucial, given the severity of Lyme disease in both dogs and humans, and is preferable to treating established infections. To effectively reduce tick infestations and the transmission of vector-borne diseases like Lyme disease, a combination of strategies is key. These include reducing tick habitats and wildlife access to yards, performing timely tick checks, applying appropriate repellent products for humans, vaccinating dogs living in tick-endemic areas, and treating pets with effective acaricidal products. The robust efficacy and sustained speed of kill of 20 mg/kg lotilaner contained in Credelio and Credelio Quattro consistently kill ticks for an entire month, effectively reducing local tick populations.

Lotilaner, a proven veterinary medicine, also shows promise in human health. It is approved as a topical treatment for *Demodex* blepharitis (Xdemvy^®^, Tarsus Pharmaceuticals, Irvine, CA, USA) and is being studied for its potential to prevent Lyme disease [38].

## Conclusions

These laboratory studies confirm that a single dose of lotilaner, administered as Credelio or Credelio Quattro (in combination with moxidectin, praziquantel, and pyrantel), was effective for the prevention of *B. burgdorferi* transmission from infected *I. scapularis* to dogs by killing ticks before transmission could occur.

## Data Availability

Data supporting the conclusions of this article are included within the article.

## Abbreviations

LSM: Least squares mean
WAAVP: World Association for the Advancement of Parasitology
